# Pregnancy Outcome Patterns of Medicaid-Eligible Women, 1999-2014: A National Prospective Longitudinal Study

**DOI:** 10.1177/2333392820941348

**Published:** 2020-07-31

**Authors:** James Studnicki, John W. Fisher, David C. Reardon, Christopher Craver, Tessa Longbons, Donna J. Harrison

**Affiliations:** 1 Charlotte Lozier Institute, Arlington, VA, USA; 2 Elliot Institute, Springfield, IL, USA; 3 American Association of Pro-Life Obstetricians and Gynecologists, Eau Claire, MI, USA

**Keywords:** pregnancy outcomes, induced abortion, natural fetal loss, Medicaid

## Abstract

**Introduction:**

The number and outcomes of pregnancies experienced by a woman are consequential determinants of her health status. However, there is no published research comparing the patterns of subsequent pregnancy outcomes following a live birth, natural fetal loss, or induced abortion.

**Objectives:**

The objective of this study was to describe the characteristic patterns of subsequent pregnancy outcomes evolving from each of three initiating outcome events (birth, induced abortion, natural fetal loss) occurring in a Medicaid population fully insured for all reproductive health services.

**Methods:**

We identified 7,388,842 pregnancy outcomes occurring to Medicaid-eligible women in the 17 states which paid for abortion services between 1999-2014. The first known pregnancy outcome for each woman was marked as the index outcome which assigned each woman to one of three cohorts. All subsequent outcomes occurring up to the fifth known pregnancy were identified. Analyses of the three index outcome cohorts were conducted separately for all pregnancy outcomes, three age bands (<17, 17-35, 36+), and three race/ethnicity groups (Hispanic, Black, White).

**Results:**

Women with index abortions experienced more lifetime pregnancies than women with index births or natural fetal losses and were increasingly more likely to experience another pregnancy with each subsequent pregnancy. Women whose index pregnancy ended in abortion were also increasingly more likely to experience another abortion at each subsequent pregnancy. Both births and natural fetal losses were likely to result in a subsequent birth, rather than abortion. Women with natural losses were increasingly more likely to have a subsequent birth than women with an index birth. All age and racial/ethnic groups exhibited the characteristic pattern we have described for all pregnancy outcomes: abortion is associated with more subsequent pregnancies and abortions; births and fetal losses are associated with subsequent births. Other differences between groups are, however, apparent. Age is positively associated with the likelihood of a birth following an index birth, but negatively associated with the likelihood of a birth following an index abortion. Hispanic women are always more likely to have a birth and less likely to have an abortion than Black or White women, for all combinations of index outcome and the number of subsequent pregnancies. Similarly, Black women are always more likely to have an abortion and less likely to experience a birth than Hispanic or White women.

**Conclusion:**

Women experiencing repeated pregnancies and subsequent abortions following an index abortion are subjected to an increased exposure to hemorrhage and infection, the major causes of maternal mortality, and other adverse consequences resulting from multiple separation events.

## Introduction

A woman’s reproductive history is a consequential determinant of her health status. The number of pregnancies experienced, and the outcomes of each pregnancy, may impact the women’s own physical and mental health, longevity, and the outcomes of future subsequent pregnancies.^1-7^ Low income women may be particularly susceptible to the possibility of any adverse effects and, therefore, the costs of state Medicaid programs may also be impacted by the differences in the number and sequence of pregnancy outcomes, as well as any resultant health services utilization.

In our search of the existing literature, we found no published research on the patterns of subsequent pregnancy outcomes following a live birth, induced abortion, or natural fetal loss. While the reproductive history of each woman is influenced by multiple interacting medical and personal circumstances, an aggregated view of the different longitudinal trajectories of subsequent pregnancy outcomes can inform both the clinical research regarding the effect of pregnancy outcomes and the policy discussion regarding public funding of reproductive health services.

The Hyde Amendment bans the use of federal funds for abortion coverage except in cases of rape, incest, or life endangerment of the mother, which represent only a tiny fraction of induced abortions. However, 17 states have had a policy to use their own Medicaid funds to provide payment for essentially all abortions. In states that fund abortion, Medicaid beneficiaries represent more than half of all abortions.^8^ This circumstance provides an opportunity to investigate the sequencing of pregnancy outcomes over an extended period of time. Therefore, the objective of this study was to describe the characteristic patterns of subsequent pregnancy outcomes evolving from each of 3 initiating outcome events (birth, induced abortion, natural fetal loss) occurring in a Medicaid population fully insured for all reproductive health services.

## Methods

Data were obtained from the enrollee-level Medicaid Analytic eXtract (MAX) licensed through the Centers for Medicare and Medicaid Services (CMS) Chronic Condition Data Warehouse’s (CCW) Medicaid data. At the time of this study data were available for years 1999 through 2014. The study population consisted of enrollees from the 17 states where Medicaid includes coverage of all reproductive health care services, including induced abortion. Due to lags in reporting timeframes not all states had submitted claims data through 2014. The last year of data relative to each of the states was 2012 for Alaska, Illinois, Maryland, Montana, and New Mexico; through 2013 for Arizona, Connecticut, Hawaii, Massachusetts, New York, Oregon, and Washington; and through 2014 for California, Minnesota, New Jersey, Vermont, and West Virginia.

The study population was limited to women over 13 years of age with at least one identifiable pregnancy outcome from 1999 through the latest year of data available for each state. During the study period all unique pregnancy outcomes were identified for each beneficiary using *International Classification of Diseases, Ninth Revision* (*ICD9*) codes. In addition, *Current Procedural Terminology, 4th Edition* (*CPT4*) and Healthcare Common Procedure Coding System (HCPCS) codes were utilized to verify pregnancy outcomes.

Based on these codes, all pregnancy outcomes were subdivided into 4 categories: live birth (*ICD9* V27.0, V27.2, and V27.5), natural fetal loss (*ICD9* V27.1, V27.4, V27.7, 630, 631, 633, 634), induced abortion (*ICD9* 635.xx, *CPT4*: 59840, 59841, 59850, 59851, 59852, 59855, 59856, 59857, and HCPCS: S0199, S2260, S2265, S2266, S2267, X7724, X7726, S0190, S0191), and undetermined (*ICD9* 636.xx, 637.xx, 638.xx). In order to uniquely define each pregnancy event, multiple diagnostic or treatment codes within 30 days of a pregnancy loss (natural, induced, or undetermined) were collapsed into a single pregnancy outcome using the first date associated with that cluster of Medicaid claims. Similarly, multiple diagnostic or treatment codes within 180 days of a delivery were collapsed into a single pregnancy outcome. Twins and multiple pregnancies resulting in a combination of both live birth and fetal loss were excluded from the analysis.

The first known pregnancy outcome for each beneficiary was marked as that woman’s index pregnancy. The index outcome is the first known pregnancy outcome for each beneficiary in this data set. At the index outcome, the beneficiary is assigned to 1 of 3 index outcome cohorts, and all subsequent outcomes occurring within the study period for pregnancies 2 to 5 are identified. The composite index cohort represents the actual summed totals of specific pregnancy outcomes in order of occurrence. Our analytical objective was to determine whether there were significant differences in the patterns of pregnancies subsequent to each of 3 index pregnancy outcomes. Since these patterns involved multiple outcomes with sequential and incremental effects, no multivariate model with a single dependent outcome could represent all dimensions of these patterns. We concluded that a comprehensive descriptive approach would enable detection of significant differences in the overall pattern and also enable comparison for each index outcome/subsequent pregnancy combination.

Tables were constructed to identify subsequent pregnancy outcomes for each index outcome cohort. A comprehensive analysis was conducted separately for all pregnancy outcomes, 3 age bands (<17, 17-35, 36+), and 3 race/ethnicity groups (Hispanic, black, white). Women were placed in age bands based upon their age at the time of the index pregnancy outcome without consideration of age at each subsequent pregnancy outcome. For all group comparisons, we calculated odds ratios (OR) and confidence intervals (CI) for *P* < .05. Summary analytic tables were created using (SAS/STAT) software, version (10) of the SAS System for (Unix). Copyright (2019) SAS Institute Inc. All comparative analyses were completed using Microsoft Excel (Version 16).

## Findings

### The Study Population

During the study period, 7 388 842 pregnancy outcomes were identified as either a live birth, natural loss, or induced abortion which occurred as the index through the fifth pregnancy (Table 1). Another 540 393 pregnancy outcomes were undetermined. With each successive pregnancy, a decreasing percent of births and an increasing percent of abortions were apparent. Births were 81.4% of the known outcomes of index pregnancies but only 51.6% of the known fifth pregnancy outcomes. Abortions, by contrast increased from 9.0% of the index pregnancies to 33.1% of fifth pregnancies. Natural losses also increased from 9.6% of the index pregnancies to 15.3% of known fifth pregnancies.

**Table 1.** Total Pregnancy Outcomes, Index and Subsequent 2 to 5 Number and Percent of Known Outcomes.

Cohort pregnancy number	Live birth	% Known	Natural loss	% Known	Induced abortion	% Known	Undetermined	Grand total
Index	3 807 694	81.40%	449 182	9.60%	421 011	9.00%	288 662	4 966 549
2	1 178 787	72.10%	208 712	12.80%	247 432	15.10%	140 527	1 775 458
3	427 171	64.80%	94 926	14.40%	136 847	20.80%	66 078	725 022
4	165 370	58.00%	42 782	15.00%	76 967	27.00%	30 388	315 507
5	68 047	51.60%	20 171	15.30%	43 743	33.10%	14 738	146 699
Grand total	5 647 069	76.40%	815 773	11.00%	926 000	12.50%	540 393	7 929 235

### Subsequent Pregnancies

**Figure 1.**
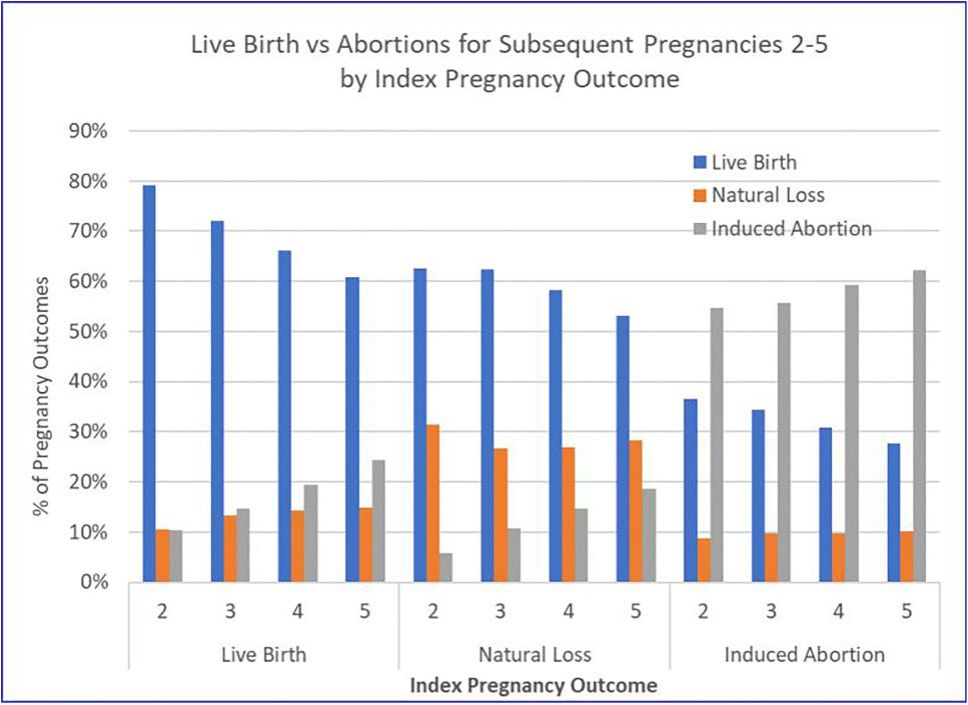
Live Birth vs. Abortions for Subsequent Pregnancies 2-5 by Index Pregnancy Outcome.

Table 2 and Figure 1 show the pattern of pregnancy outcomes for each index pregnancy cohort. Women whose index pregnancy was an induced abortion experienced more subsequent pregnancies than women with other index pregnancy outcomes. The 421 011 women who had index pregnancy abortions experienced 425 814 known outcomes of subsequent pregnancies 2 to 5, or 101% of the number of index pregnancies. Women with index pregnancy births and natural losses had significantly fewer subsequent total pregnancies 2 to 5 (2 167 955; 56.9% and 368 917; 82.1%, respectively). Women with index pregnancy abortions were also increasingly more likely to experience a pregnancy following each subsequent pregnancy than were women with index pregnancy births: second pregnancy OR: 1.84, CI (1.83-1.85); third pregnancy OR: 2.36, CI (2.35-2.38); fourth pregnancy OR: 3.04, CI (3.01-3.07); fifth pregnancy OR: 3.86, CI (3.81-3.91).

**Table 2.** Total Pregnancy Outcomes for Index and Subsequent Pregnancies, Number and Percent of Pregnancies 2 to 5, and the Index Outcome Cohort.

Index pregnancy live birth	Live birth	% of Known	Natural loss	% of Known	Induced abortion	% of known	Undetermined	Total	% of Cohort (Table 1)
2nd Pregnancy	986 834	79.1%	131 302	10.5%	129 168	10.4%	99 587	1 346 891	35.4%
3rd Pregnancy	339 676	72.0%	62 704	13.3%	69 423	14.7%	45 936	517 739	13.6%
4th Pregnancy	126 645	66.2%	27 470	14.4%	37 086	19.4%	20 241	211 442	5.6%
5th Pregnancy	50 294	60.9%	12 279	14.9%	20 069	24.3%	9241	91 883	2.4%
Total	1 503 449	75.4%	233 755	11.7%	255 746	12.8%	175 005	2 167 955	56.9%
Index pregnancy natural loss	Live birth	% of Known	Natural loss	% of Known	Induced abortion	% of Known	Undetermined	Total	% of cohort (Table 1)
2nd Pregnancy	120 189	62.7%	60 305	31.5%	11 220	5.9%	25 670	217 384	48.4%
3rd Pregnancy	51 326	62.4%	22 020	26.8%	8921	10.8%	10 881	93 148	20.7%
4th Pregnancy	20 710	58.3%	9570	27.0%	5219	14.7%	4731	40 230	9.0%
5th Pregnancy	8515	53.1%	4540	28.3%	2978	18.6%	2122	18 155	4.0%
Total	200 740	61.7%	96 435	29.6%	28 338	8.7%	43 404	368 917	82.1%
Index pregnancy induced abortion	Live birth	% of Known	Natural loss	% Known	Induced abortion	% of Known	Undetermined	Total	% of Cohort (Table 1)
2nd Pregnancy	71 764	36.6%	17 105	8.7%	107 044	54.6%	15 270	211 183	50.2%
3rd Pregnancy	36 169	34.5%	10 202	9.7%	58 503	55.8%	9 261	114 135	27.1%
4th Pregnancy	18 015	30.8%	5742	9.8%	34 662	59.3%	5416	63 835	15.2%
5th Pregnancy	9238	27.8%	3352	10.1%	20 696	62.2%	3375	36 661	8.7%
Total	135 186	34.4%	36 401	9.3%	220 905	56.3%	33 322	425 814	100.1%
Subsequent pregnancy totals	1 839 375	67.8%	366 591	13.5%	504 989	18.6%	251 731	2 962 686	59.7%

### Subsequent Pregnancy Outcomes

Women whose index pregnancy ended in abortion had more abortions than any other pregnancy outcome for subsequent pregnancies 2 to 5; and they increasingly selected abortion over birth with each subsequent pregnancy, as shown in Table 2 and Figure 1: second pregnancy OR: 1.49, CI (1.48-1.51); third pregnancy OR: 1.62, CI (1.60-1.64); fourth pregnancy OR: 1.92, CI (1.89-1.96); fifth pregnancy OR: 2.24, CI (2.19-2.30).

Women who had index pregnancy births had more births than any other known pregnancy outcome for subsequent pregnancies 2 to 5, but abortion did become more likely with each subsequent pregnancy.

The 3 807 694 women who had index pregnancy births consistently but decreasingly selected birth rather than abortion for subsequent outcomes: second pregnancy OR: 7.64, CI (7.59-7.69); third pregnancy OR: 4.89, CI (4.85-4.93); fourth pregnancy OR: 3.41, CI (3.38-3.45); fifth pregnancy OR: 2.51, CI (2.47-2.55).

Women who had experienced index natural losses (n = 449 182) were increasingly more likely than index birth women to have a live birth in subsequent pregnancies: second pregnancy OR: 2.39, CI (2.36-2.41); third pregnancy OR: 2.68, CI (2.65-2.72); fourth pregnancy OR: 3.57, CI (3.50-3.64); fifth pregnancy OR: 4.52, CI (4.40-4.65).

### Age and Subsequent Pregnancy Outcomes

**Figure 2.**
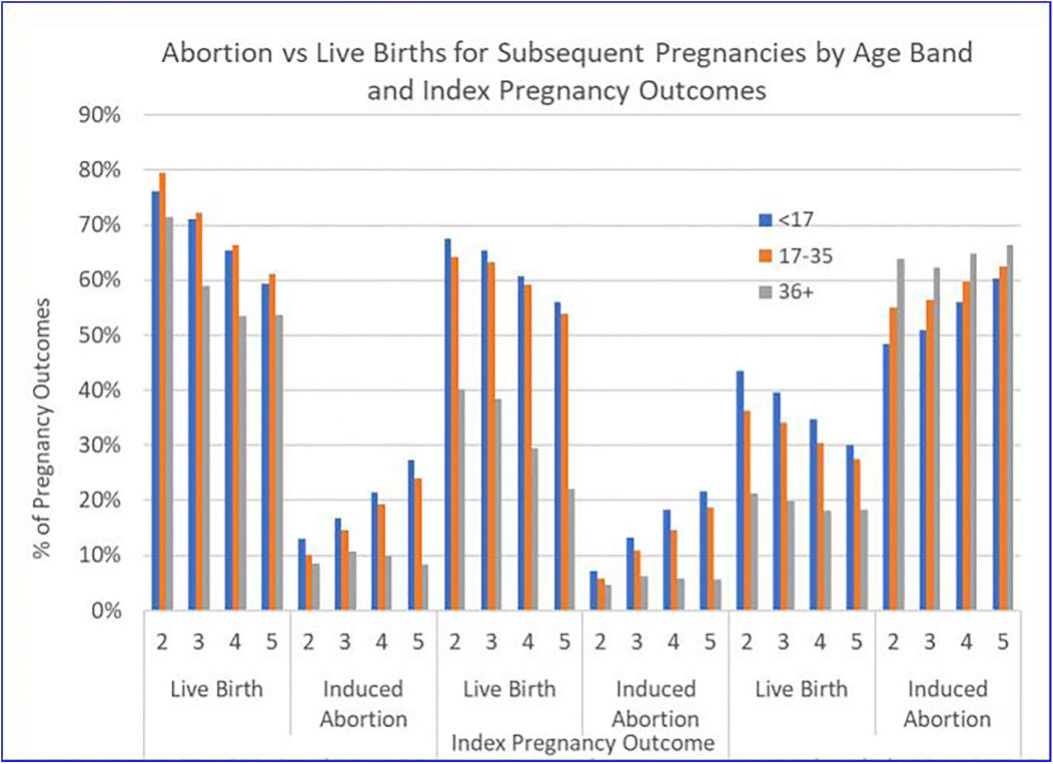
Abortion vs. Live Births for Subsequent Pregnancies by Age Band and Index Pregnancy Outcomes.

All 3 age bands (<17, 17-35, 36+) exhibited the characteristic patterns of post index pregnancy outcomes we have previously described for the entire study population of pregnancy outcomes (see Table 3 and Figure 2). That is, the abortion of an index pregnancy was associated with an increased likelihood of a subsequent abortion, which increased with each pregnancy, and the decreasing likelihood of a birth. Index births and fetal losses were associated with a greater likelihood that subsequent pregnancies would end in a birth, though the likelihood of abortion increased and the likelihood of a birth decreased with each subsequent pregnancy.

**Table 3.** Total Pregnancy Outcomes for Index and Subsequent Outcomes 2 to 5, by 3 Age Bands.

Pregnancy outcomes—index and subsequent	Age					
	<17		17-35		36+	
	Number	% Known	Number	% Known	Number	% Known
Live birth	130 267		1 605 946		22 982	
Live birth	106 552		1 376 584		20 313	
2	59 097	76.2%	911 226	79.5%	16 511	71.5%
3	28 618	71.0%	308 247	72.2%	2811	59.0%
4	13 139	65.5%	112 773	66.4%	733	53.5%
5	5698	59.3%	44 338	61.1%	258	53.8%
Abortion	23 715	78.4%	229 362	68.0%	2669	37.7%
2	10 052	13.0%	117 138	10.2%	1978	8.6%
3	6742	16.7%	62 167	14.6%	514	10.8%
4	4299	21.4%	32 650	19.2%	137	10.0%
5	2622	27.3%	17 407	24.0%	40	8.3%
Induced abortion	40 866		307 903		7322	
Live birth	17 719		115 676		1791	
2	8757	43.5%	61 718	36.4%	1289	21.2%
3	4951	39.6%	30 864	34.1%	354	19.8%
4	2636	34.7%	15 269	30.4%	110	18.2%
5	1375	30.0%	7825	27.5%	38	18.3%
Abortion	23 147	215.8%	192 227	233.6%	5531	257.3%
2	9764	48.5%	93 393	55.0%	3887	63.9%
3	6352	50.9%	51 038	56.3%	1113	62.2%
4	4267	56.1%	30 002	59.8%	393	64.9%
5	2764	60.3%	17 794	62.4%	138	66.3%
Natural loss	17 906		203 064		8108	
Live birth	15 194		178 367		7179	
2	7882	67.5%	106 897	64.2%	5410	40.2%
3	4262	65.4%	45 738	63.3%	1326	38.4%
4	2057	60.6%	18 315	59.2%	338	29.4%
5	993	55.9%	7417	53.8%	105	22.0%
Abortion	2712	60.4%	24 697	50.0%	929	22.3%
2	847	7.3%	9755	5.9%	618	4.6%
3	862	13.2%	7841	10.8%	218	6.3%
4	619	18.2%	4534	14.6%	66	5.7%
5	384	21.6%	2567	18.6%	27	5.7%

Women under the age of 17 who experience an index birth are always more likely to experience another birth rather than an abortion for subsequent pregnancies 2 to 5: second OR: 6.56, CI (6.32-6.79); third pregnancy OR: 5.45, CI (5.20-5.70); fourth pregnancy OR: 4.95, CI (4.66-5.25); fifth pregnancy OR: 4.37, CI (4.03-4.73). At the second pregnancy, they are 5.8 times more likely to have a birth than an abortion. By the fifth pregnancy, they are 2.2 times as likely.

Women ages 17 to 35 who experience an index birth are similarly more likely to experience another birth rather than an abortion at any subsequent pregnancy: second pregnancy OR: 11.77, CI (11.63-11.91); third pregnancy OR: 8.20, CI (8.07-8.33); fourth pregnancy OR: 6.79, CI (6.65-6.03); fifth pregnancy OR: 5.79, CI (5.64-5.95). At the second pregnancy, they are 7.9 times more likely to have a birth than an abortion. By the fifth pregnancy, they are 2.5 times as likely.

Women 36+ who experience an index birth are also more likely to experience another birth rather than an abortion at any subsequent pregnancy: second pregnancy OR: 25.17, CI (23.27-27.22); third pregnancy OR: 17.19, CI (15.12-19.55); fourth pregnancy OR: 19.12, CI (15.39-23.74); fifth pregnancy OR: 23.42, CI (16.31-33.64). At second pregnancy, they are 8.3 times more likely to have a birth than an abortion. By the fifth pregnancy, they are still 6.6 times as likely.

Therefore, following an index birth, age is positively associated with the likelihood of a birth. That is, women 36+ are the most likely to select birth rather than abortion for subsequent pregnancies. However, following an index abortion, age is negatively associated with the likelihood of a subsequent birth. Women 36+ are increasingly less likely to experience a birth after an index abortion, and more likely to abort any subsequent pregnancy.

Following an index natural loss, women <17 were increasingly more likely to have a birth at each subsequent pregnancy than women 36+: second pregnancy OR: 3.10, CI (2.94-3.26); third pregnancy OR: 3.04, CI (2.79-3.31); fourth pregnancy OR: 3.70, CI (3.20-4.28); fifth pregnancy OR: 4.50, CI (3.55-5.70). Note for all age groups and for all subsequent pregnancies the strong preference for a live birth rather than an abortion following a natural fetal loss. For the second pregnancy, women <17 were 9.2 times (OR: 26.59, CI: 24.55-28.81) more likely to have a birth than an abortion. Women 17 to 35 were 10.9 times as likely (OR: 28.79, CI: 28.14-29.46), and women 36+ were 8.7 times as likely (OR: 13.96, CI: 12.79-15.25).

### Race and Subsequent Pregnancy Outcomes

All racial and ethnic groups exhibited the same trend in pregnancy outcome patterns described above. Specifically, an index birth predicted more birth outcomes in subsequent pregnancies whereas an index abortion predicted more subsequent abortions. However, some racial and ethnic differences were observed.

**Figure 3.**
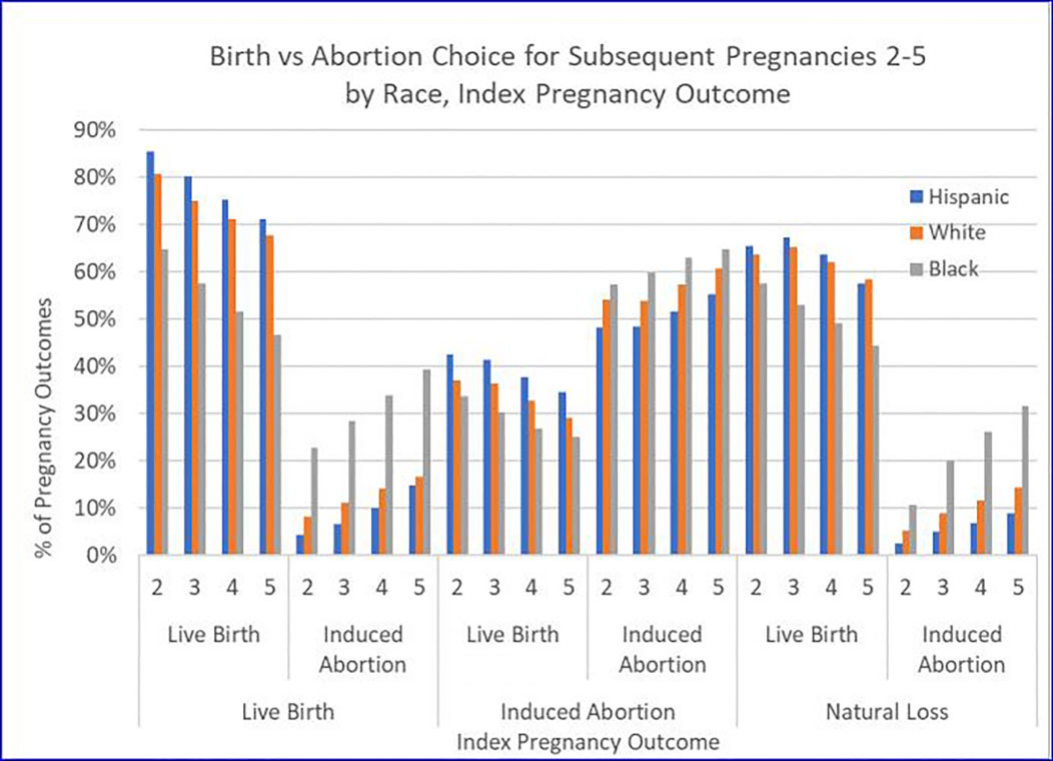
Births vs. Abortion Choice for Subsequent Pregnancies 2-5 by Race, Index Pregnancy Outcome.

As shown in Table 4 and Figure 3, the distribution of outcomes relative to the index pregnancy had significant racial differences. Hispanic women had the highest percent of index births (86.8%) and the lowest percent of index abortions (3.4%). Black women had the index lowest percent of births (72.3%) and the highest percent of index abortions (16.8%).

**Table 4.** Total Pregnancy Outcomes for Index and Subsequent Outcomes 2 to 5, by Race.

Pregnancy outcomes—index and subsequent	Race					
	Hispanic number	% Known	White Number	% Known	Black number	% known
Live birth	1 073 021	81.9%	1 397 065	76.7%	637 464	68.0%
Live birth						
2	304 817	85.5%	381 585	80.7%	155 708	65.0%
3	99 824	80.4%	135 117	75.0%	63 163	57.6%
4	33 379	75.3%	51 910	71.3%	27 310	51.7%
5	11 869	71.3%	21 223	67.8%	12 219	46.7%
Abortion						
2	15 279	4.3%	39 463	8.3%	54 392	22.7%
3	8301	6.7%	20 220	11.2%	31 224	28.5%
4	4448	10.0%	10 301	14.1%	17 954	34.0%
5	2470	14.8%	5224	16.7%	10 331	39.5%
Induced abortion	154 871	8.5%	154 871	8.5%	148 375	15.8%
Live birth						
2	9117	42.5%	25 391	37.2%	27 208	33.8%
3	4909	41.4%	12 549	36.5%	14 412	30.3%
4	2529	37.8%	5841	32.8%	7776	27.0%
5	1295	34.6%	2746	29.1%	4392	25.0%
Abortion						
2	10 353	48.2%	37 024	54.2%	46 071	57.3%
3	5751	48.5%	18 555	53.9%	28 515	59.9%
4	3451	51.6%	10 230	57.4%	18 190	63.1%
5	2075	55.4%	5751	60.9%	11 417	64.9%
Natural loss	165 214	9.1%	165 214	9.1%	95 832	10.2%
Live birth						
2	32 686	65.6%	46 706	63.8%	24 740	57.6%
3	13 893	67.3%	20 813	65.3%	10 703	53.0%
4	5315	63.7%	8510	62.1%	4741	49.1%
5	2039	57.6%	3534	58.5%	2164	44.3%
Abortion						
2	1301	2.6%	3873	5.3%	4640	10.8%
3	1026	5.0%	2814	8.8%	4053	20.1%
4	574	6.9%	1583	11.5%	2537	26.3%
5	315	8.9%	871	14.4%	1550	31.7%

Following a live birth index outcome, Hispanic women were always more likely to experience a subsequent birth and less likely to experience a subsequent abortion than white or black women. Hispanic women were 21.2 times (OR: 132.03, CI: 129.58-134.52) more likely to have a birth rather than an abortion for a second subsequent pregnancy following an index birth. Black women who had an index birth were 2.9 times (OR: 6.32, CI: 6.24-6.40) more likely to experience a birth rather than abortion.

Following an index abortion, black women were more likely than white and Hispanic women to have an induced abortion and less likely to have a live birth for subsequent pregnancies 2 to 5. As always, the likelihood of an abortion increased with every subsequent pregnancy.

Following an index natural loss, similar to the pattern following an index live birth, live birth was preferred overwhelmingly to abortion for subsequent pregnancies. Even here, however, the high prevalence of abortion for black women was noteworthy. For subsequent pregnancies 2 to 5, and following an index natural loss, black women were 4.1 times (OR: 4.52, CI: 4.24-4.81), 4.1 times (OR: 4.80, CI: 4.48-5.15), 3.8 times (OR: 4.82, CI: 4.40-5.28), and 3.6 times (OR: 4.76, CI: 4.23-5.27) more likely to experience an abortion than Hispanic women.

## Discussion

Abortion is associated with a starkly different pattern of subsequent pregnancy outcomes as compared to the pattern that is more common following live birth or natural fetal loss. Moreover, abortion is associated with an increase in the total number of pregnancies a woman will experience, and those pregnancies are more likely to result in subsequent abortions. Births and natural fetal losses, by contrast, are more likely to be followed by subsequent births.

Experiencing an index abortion is associated with an increase in the total number of pregnancies experienced by a woman in her lifetime. Each pregnancy involves the separation of the mother and the embryo/fetus. Maternal mortality encompasses live birth mortality but also mortality from all other pregnancy outcomes including induced abortion.^9^ Maternal deaths from hemorrhage and infection, the major causes of maternal mortality worldwide, occur at the time of the separation event. The more frequent the separation events, the more often a woman is exposed to risk. The hope that easier access to abortion would decrease maternal mortality has been rebutted by evidence to the contrary.^10-12^ These findings demonstrating that low-income women who have abortions are more likely to have more overall pregnancies, including more subsequent abortions, would tend to support the body of evidence indicating that abortion is associated with greater subsequent reproductive health risks.^13,14^

Abortion decreases the likelihood of a birth in any subsequent pregnancy. More than 60% of surveyed women seeking an abortion indicate that they had not completed their childbearing.^15^ Yet our findings show that abortion decreases the likelihood of a birth in the immediate subsequent pregnancy. This finding is inconsistent with the idea that abortion is primarily used to optimize child spacing. Instead, our finding supports the view that women who have experienced abortions may be more likely to seek replacement pregnancies, consciously or unconsciously, which may then result in more subsequent abortions because the pressures leading to the initial abortion remain unresolved.^16^ Repeat abortions may also be evidence that some women undergo multiple abortions as a form of self-punishment.^16^ Conflicting feelings over becoming pregnant (on one hand, a desire for a replacement pregnancy; on the other, a desire to avoid another pregnancy) may lead to more risk-taking behavior in the form of irregular birth control practices and reliance on less reliable methods of contraception. This pattern is suggested by a study regarding the advance provision of emergency contraception (EC) which found that over 65% of young women reported inconsistent use of birth control and that those provided with EC took even more risks relative to their birth control options.^17^ While the correlates and determinants of these patterns are subject to alternative explanations, the pregnancy outcome trajectory described here is clear: abortion begets abortion.

Support for public funding of abortion is partly based upon the assumption that it will enable women to continue their education and careers and stabilize their personal relationships, thus enabling a happier and healthier life. A cascade of repetitive pregnancies and abortions, however, is likely to subject a woman to the various adverse effects associated with these outcomes.

There are limitations related to the use of Medicaid claims data. Medicaid-eligible beneficiaries are by definition financially disadvantaged and are not representative of all women experiencing abortion. Conversely, a data set composed entirely of low-income women may also be considered an advantage since results are unlikely to be explained by differences in income or other factors strongly associated with income. Services received by eligible women but paid by another source (eg, out of pocket) are not included in the claims data. Services received when the women were not eligible are similarly not included. Administrative data are also subject to limitations regarding coding errors, inconsistent coding, and the exclusion of codes considered nonessential for billing. ^18,19^ There are inconsistencies in coding which may vary by state. Our data extraction protocol required an *ICD* code to identify beneficiaries who had an induced abortion. To the extent that some states or individual providers do not code an abortion with an *ICD* code, our study population may undercount the number of abortions. This undercount would likely be due to a random variation in coding protocols and is unlikely to affect the trends related in our findings.

The findings described here are also a prelude to future explanatory analyses, utilizing multivariate methods, which enable a more granular understanding of these outcomes. For example, the interpregnancy time interval associated with the index events may be influenced by sociodemographic factors as well as pre-index outcome health services utilization. Similarly, comparison of subpopulations of interest defined by this analysis can be addressed with multivariate methods, such as the demographic and regional differences in populations of Medicaid women without births versus those without abortions.

The results of this study suggest that Medicaid funding of abortion may have a direct impact on the number and outcome of subsequent pregnancies. Additional research is necessary to examine the impact of pregnancy outcome differences on total health care utilization, including hospitalizations, emergency room visits, prescription drug history, and psychiatric and behavioral services. Further research into this domain is essential.
